# AXDND1, a novel testis-enriched gene, is required for spermiogenesis and male fertility

**DOI:** 10.1038/s41420-021-00738-z

**Published:** 2021-11-11

**Authors:** Qian Ma, Congcong Cao, Changshui Zhuang, Xiaomin Luo, Xiaofeng Li, Huijuan Wan, Jing Ye, Fangfang Chen, Lina Cui, Yan Zhang, Yujiao Wen, Shuiqiao Yuan, Yaoting Gui

**Affiliations:** 1 Guangdong Key Laboratory of Male Reproductive Medicine and Genetics, Institute of Urology, Peking University Shenzhen Hospital, Shenzhen PKU-HKUST Medical Center, Shenzhen, Guangdong 518036 China; 2 Institute of Reproductive Health, Tongji Medical College, Huazhong University of Science and Technology, Wuhan, Hubei 430030 China; 3 Shenzhen Huazhong University of Science and Technology Research Institute, Shenzhen, Guangdong 518057 China; 4 Laboratory Animal Center, Huazhong University of Science and Technology, Wuhan, Hubei 430030 China

**Keywords:** Spermatogenesis, Infertility

## Abstract

Spermiogenesis is a complex process depending on the sophisticated coordination of a myriad of testis-enriched gene regulations. The regulatory pathways that coordinate this process are not well understood, and we demonstrate here that AXDND1, as a novel testis-enriched gene is essential for spermiogenesis and male fertility. AXDND1 is exclusively expressed in the round and elongating spermatids in humans and mice. We identified two potentially deleterious mutations of AXDND1 unique to non‐obstructive azoospermia (NOA) patients through selected exonic sequencing. Importantly, *Axdnd1* knockout males are sterile with reduced testis size caused by increased germ cell apoptosis and sloughing, exhibiting phenotypes consistent with oligoasthenoteratozoospermia. *Axdnd1* mutated late spermatids showed head deformation, outer doublet microtubules deficiency in the axoneme, and loss of corresponding accessory structures, including outer dense fiber (ODF) and mitochondria sheath. These phenotypes were probably due to the perturbed behavior of the manchette, a dynamic structure where AXDND1 was localized. Our findings establish AXDND1 as a novel testis-enrich gene essential for spermiogenesis and male fertility probably by regulating the manchette dynamics, spermatid head shaping, sperm flagellum assembly.

## Introduction

Spermiogenesis is the last phase of spermatogenesis, during which haploid round spermatids undergo a series of morphological changes to become elongated spermatids. Specifically, the Golgi-derived acrosome anchors to the anterior membrane of the nucleus via the acroplaxome plate, while the microtubule- and F-actin scaffolded manchette girdles the posterior pole of the nucleus [1–4]. Under the synergistic effects of the acrosome-acroplaxome, the manchette, and the ectopic speciation of Sertoli cells, species-specific head shape of spermatozoan is formed accordingly [5]. Meanwhile, the sperm tail grows from the centrioles at the basal body with the help of the intra-manchette protein transport (IMT) system as well as the intra-flagellar transport (IFT) system driven by motor proteins [6]. These events are well orchestrated, and any step going wrong might lead to malformed spermatids with head or tail defects, and ultimately, male infertility.

Manchette is a transient skirt structure surrounding the head of spermatids, which appears at step 8, and disassembles at step 14 spermatids during spermatid elongation in mice [6, 7]. Its assembly, dynamic movement, and its mediated protein transport system are of great importance in spermiogenesis [7]. Many IFT proteins located in the manchette and were involved in sperm tail formation during spermiogenesis [8]. For instance, in the *Ift88* mutant sperm, axonemal ODF (outer dense fiber) proteins were accumulated in the manchette, which suggested that IMT is essential for the transport of ODF protein [9]. KIF3A-deficiency in mice resulted in sperm head deformation and disrupted transport of cargo proteins involved in sperm tail assemblies, such as MNS1, ODF3, and CBE1, which were abnormally accumulated in the manchette than being correctly assembled in the sperm tail [10, 11]. HOOK protein could bind to dynein or subunits of kinesin such as KLC3, act as an adaptor, and mediate the transport of cargo protein LRGUK1 and RIMBP3 along the manchette [12–15]. Although great progress has been made during the last decades on details of spermiogenesis, the mechanisms underlying are still far from being fully understood.

Human *AXDND1* (axonemal dynein light chain domain containing 1) gene is located in Chromosome 1 and has 24 exons encoding a 118 KD protein. Mouse *Axdnd1* is also situated in Chromosome 1 and is composed of 25 exons. According to expression profiles on the NCBI website (https://www.ncbi.nlm.nih.gov/gene/?term=axdnd1), these two genes are highly enriched in the testicular tissue of both humans and mice. Bioinformatic analysis showed that AXDND1 contains a conserved axonemal dynein light chain domain (pfam10211). It was reported that the axonemal dynein light chain could regulate the cilia motility via direct binding to the N-terminal domain of the heavy chain, which suggests that AXDND1 might be involved in sperm motility [16, 17]. However, no information about the expression and function of AXDND1 has been reported yet.

In this study, we, for the first time, reported that AXDND1 is exclusively expressed in the round and late spermatids in both humans and mice and is essential for spermiogenesis and male fertility. Two potentially deleterious heterozygous mutations of *AXDND1* unique to non‐obstructive azoospermia (NOA) patients were found through selected exonic sequencing. *Axdnd1* mutant male mice were infertile with reduced testis size caused by increased germ cell apoptosis and sloughing. Testicular late spermatids were deformed with abnormal manchette structure. Our studies demonstrate that AXDND1 regulates the manchette dynamics, spermatid head shaping, sperm flagellum assembly, and ultimately male fertility.

## Results

### AXDND1 may play an important role in human spermatogenesis

**Fig. 1:**
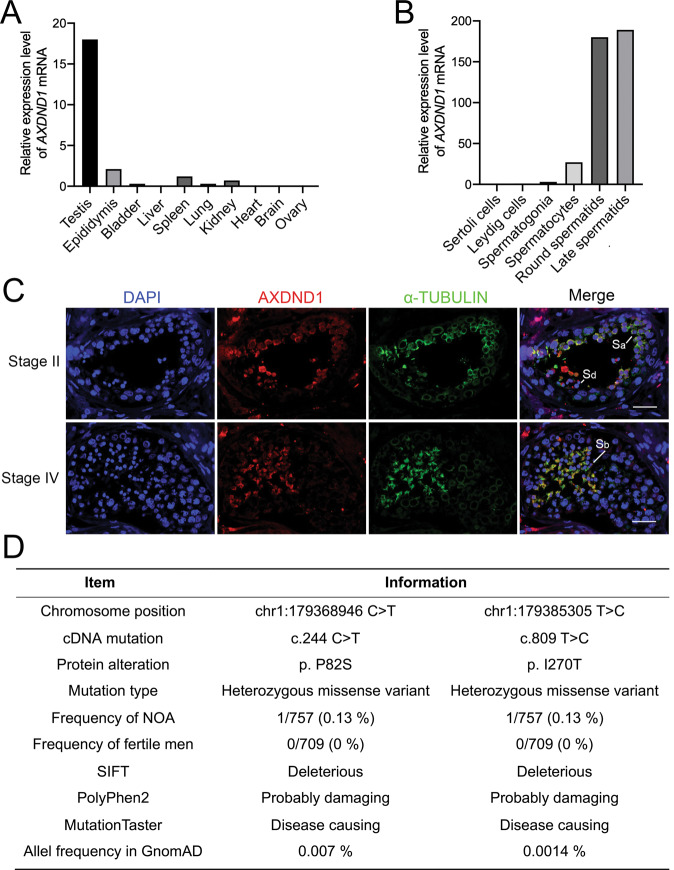
The expression characteristics of *AXDND1* in human testis and clinical mutation screening. **A** *AXDND1* mRNA expression in various human tissues. **B***AXDND1* expression in different types of human testicular cells. Data in (**A**) and (**B**) were obtained from the Human Protein Atlas. **C** AXDND1 protein expression in the human testis was determined by co-immunofluorescent staining of AXDND1 (red) and α-TUBULIN (green). AXDND1 was highly expressed and colocalized with α-TUBULIN in round spermatids (Sa) in stage II and late spermatids (Sb) in stage IV. Scale bar: 20 µm. **D** Analysis of AXDND1 variations in NOA patients.

Through analyzing high-throughput gene expression profiles of human tissues, cell lines, as well as single-cell expression profiles reported at the Human Protein Atlas (https://www.proteinatlas.org/), we found that *AXDND1* is a testis-enriched gene (Fig. 1A), and highly expressed in the round and late spermatids of human (Fig. 1B). Immunofluorescent staining also showed that *AXDND1* was mainly located in the cytoplasm of round and late spermatids (Fig. 1C). To determine whether AXDND1 is associated with clinical azoospermia, we conducted a large-scale exonic sequence analysis of the *AXDND1* gene from our previous published data containing 757 NOA patients and 706 fertile men [18]. Strikingly, we found 9 missense mutations and 18 intronic variations of *AXDND1* unique to NOA patients (Supplementary Table S1), among which two variations (p.P82S, p.I270T) were predicted to be deleterious by SIFT, PolyPhen2 and MutationTaster (Fig. 1D). The NOA patients harboring p.I270T mutation also carried a p.T624M variation, but p.T624M mutation was predicted benign. No homozygous mutations unique to NOA patients had been identified, indicating recessive AXDND1 may relate to NOA, and the deleterious heterozygous mutation of AXDND1 might increase the risk of NOA. These results suggested that AXDND1 may have a function on human spermatogenesis and male fertility.

### *Axdnd1* encodes a highly conserved protein expressed exclusively in mouse testes

**Fig. 2:**
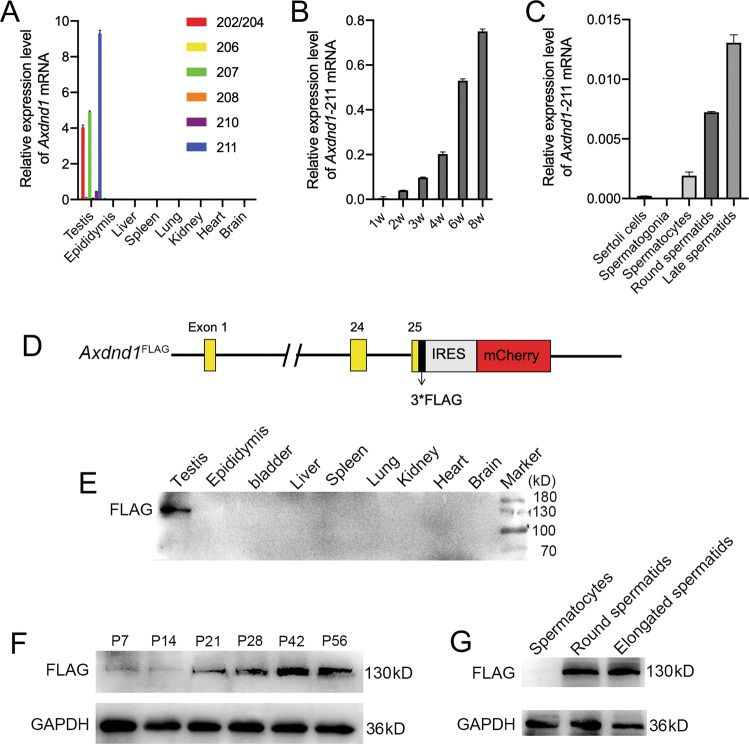
The expression pattern of AXDND1 in mouse testis. **A** The expression profile of different isoforms of *Axdnd1* transcripts in various mouse tissues is analyzed by RT-qPCR. **B** Expression of the most enriched full-length *Axdnd1* transcript (*Axdnd1*-211) in the testes of mice aged 1, 2, 3, 4, 6, and 8 weeks. **C** Relative mRNA levels of *Axdnd1*-211 in isolated mouse testicular germ cells. **D** The schematic of the generation of a mouse model expressing FLAG-tagged AXDND1 is shown. **E**–**G** Western blot analyses of AXDND1 expression in multiple tissues (**b**), developmental testes (**F**), and isolated germ cells (**G**) from *Axdnd1*^FLAG/FLAG^ male mice are shown. GAPDH served as a loading control.

Multi-alignment analyses of AXDND1 orthologs in six mammalian species revealed that AXDND1 is highly conserved during evolution (Supplementary Fig. S1). Human AXDND1 shares 65% amino acid sequences with its orthologs in mice, suggesting a conserved function of AXDND1 between humans and mice. We thus examined the expression pattern of the *Axdnd1* gene in mice. Consistent with the expression of AXDND1 in humans, multi-tissue RT-qPCR analysis showed that *Axdnd1* was exclusively expressed in mouse testes (Fig. 2A), with the full-length transcript (*Axdnd1*-211) being the most abundant one among seven transcripts (Fig. 2A). The time-course expression profile of *Axdnd1*-211 was evaluated using postnatal testes from mice of different ages. Data showed that *Axdnd1* began to express at P14 and then increase in an age-dependent manner (Fig. 2B). We then isolated testicular germ cells through STA-PUT and found *Axdnd1*-211 was mainly expressed in the round and late spermatids (Fig. 2C).

To further explore the localization of AXDND1 protein in mouse testis, we tried almost all commercial antibodies and customized antibodies bought from the company, yet no one worked. Then we generated a mouse model expressing FLAG-tagged AXDND1 (Fig. 2D). Western blot results using FLAG antibody showed that a testis-specific band at 130 kDa was observed, consistent with the theoretical molecular weight of mouse AXDND1 (Fig. 2E). Consistent with mRNA expression, AXDND1 protein starts to express at P21 and then increases in an age-dependent manner (Fig. 2F). Further Western blot analyses of isolated testicular germ cells revealed AXDND1 is expressed in the round and elongated spermatids (Fig. 2G), which is also consistent with its mRNA expression. However, when applied to immunofluorescent staining, no signal could be obtained when FLAG antibody was used to detect AXDND1. Together, the above results showed that *Axdnd1* encodes a highly conserved protein expressed specifically in testes, suggesting it play an essential role in late spermatogenesis.

### *Axdnd1* gene knockout in mice results in male infertility

**Fig. 3:**
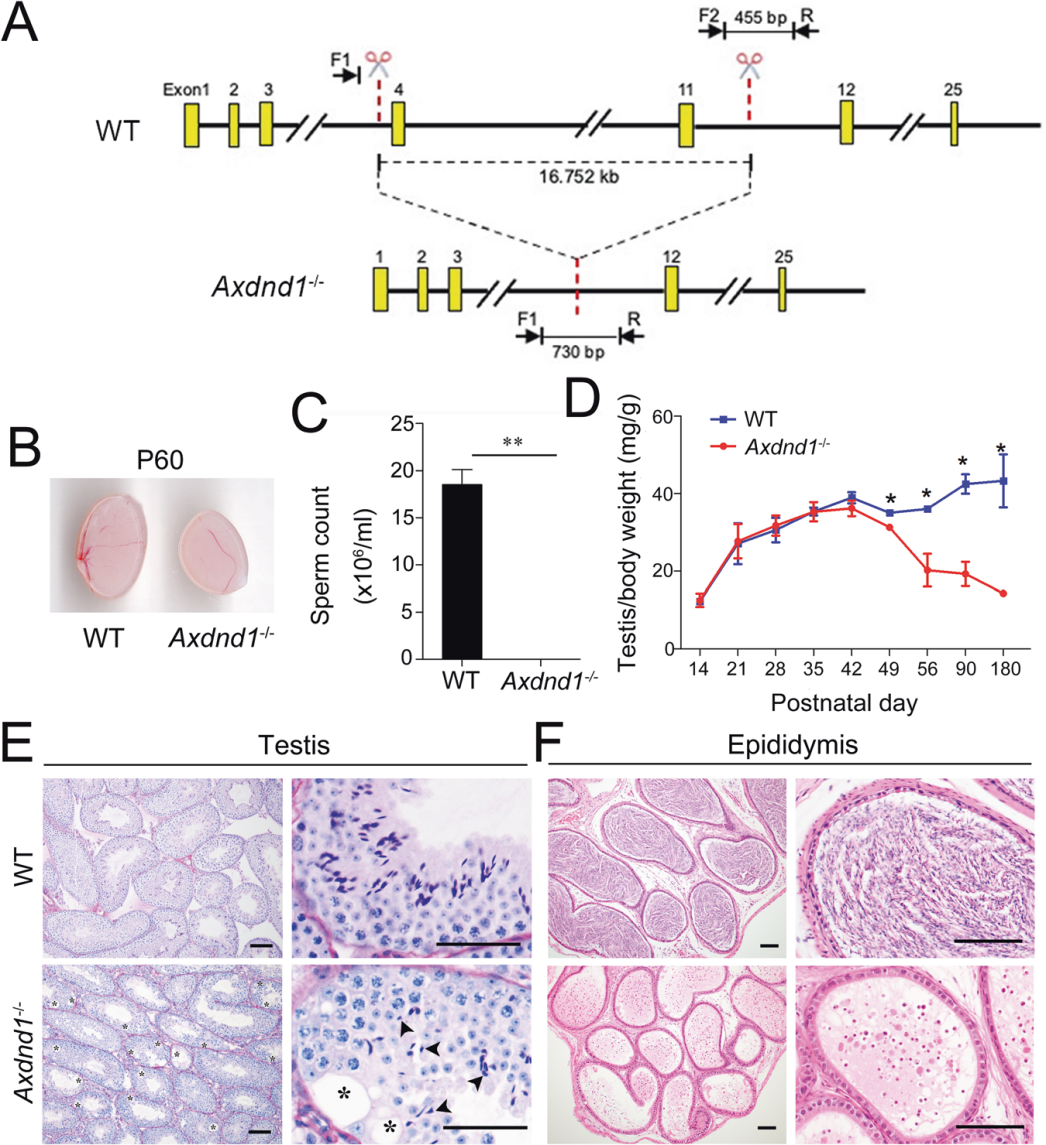
*Axdnd1*^*−/−*^ male mice are infertile with small testis size and abnormal spermiogenesis. **A** The strategy used to generate *Axdnd1* knockout mouse model via CRISP/Cas9 technology. A large fragment (16.752 kb) was deleted in *Axdnd1*^*−/−*^ mice. **B** Gross morphology of testes from wild type (WT) and *Axdnd1*^*−/−*^ male mice at postnatal day 60 (P60). **C** Sperm counts analysis in caudal epididymis. Mature sperm was hardly found in the caudal epididymis of *Axdnd1*^*−/−*^ mice. Data are presented as Mean ± SEM, *n* = 6. **P < 0.01 by Student’s *t*-test. **D** Testis growth curve (ratio of the testis to body weight) in WT and *Axdnd1*^*−/−*^ mice. The testis of *Axdnd1*^*−/−*^ mice began to be smaller than WT at P49. Data are presented as Mean ± SEM, n = 3. *P < 0.05. **E**–**F** Histological analysis of the testis (**E**) and caudal epididymis (**F**) from WT and *Axdnd1*^*−/−*^ mice. Vacuoles (labeled by asterisks) and ill-shaped nuclei (labeled by black arrowheads) were observed in the seminiferous tubules of *Axdnd1*^*−/−*^ mice. Scale bar: 100 µm.

To study the physiological role of AXDND1 in spermatogenesis, we generate an *Axdnd1* global gene knockout mouse model (*Axdnd1*^*−/−*^) using the CRISPR/Cas9 strategy (Fig. 3A). Two *Axdnd1*^*−/−*^ lines were obtained, with 16752 bp and 16760 bp depleted, respectively (Supplementary Fig. S2A, B). As the phenotype was the same between these two lines of *Axdnd1*^*−/−*^ mice, we refer to both as *Axdnd1*^*−/−*^ mice other than specifically noted. Fecundity test showed that *Axdnd1*^*−/−*^ male mice were sterile, while fertility of *Axdnd1*^*−/−*^ female mice was not affected (Table 1). As is shown in Fig. 3B, compared to the wild-type (WT) mice, the testis size of adult *Axdnd1*^*−/−*^ male mice was significantly reduced. The number of spermatozoa retrieved from adult *Axdnd1*^*−/−*^ cauda epididymis was dramatically reduced compared with WT controls (Fig. 3C). Moreover, the testis growth curve analyses showed that the ratio of the testis to body weight of *Axdnd1*^*−/−*^ male mice were smaller than WT mice at P49 onwards (Fig. 3D). Further PAS staining revealed that many vacuoles and some ill-shaped nuclei of elongating spermatids could be found in the seminiferous tubules of *Axdnd1*^*−/−*^ mouse testes (Fig. 3E). H&E staining of caudal epididymis showed no crescent-shaped mature sperm, but many cells with round nuclei existed, suggesting an extremely low number of mature sperm (Fig. 3F). Immunofluorescent staining of the germ cell marker, DDX4, revealed that most cells in caudal epididymis were immature DDX4-positive germ cells (Supplementary Fig. S2C), which were probably sloughed from testicular tubules with vacuoles. Together, these data indicated that *Axdnd1* is required for spermatogenesis and male fertility in mice.

**Table 1:** The fecundity test of *Axdnd1*^*−/−*^ mice.

*Axdnd1* genotype		Number of female mice	Litter size
Male	Female		
+/+	+/+	10	8.0 ± 2.3
*Axdnd1*^–/–^	+/+	6	0
*Axdnd1*^+/–^	*Axdnd1*^–/–^	12	7.1 ± 1.8
+/+	*Axdnd1*^–/–^	6	7.6 ± 2.1

### Increased loss and apoptosis of late spermatids in *Axdnd1*^−/−^ mice

**Fig. 4:**
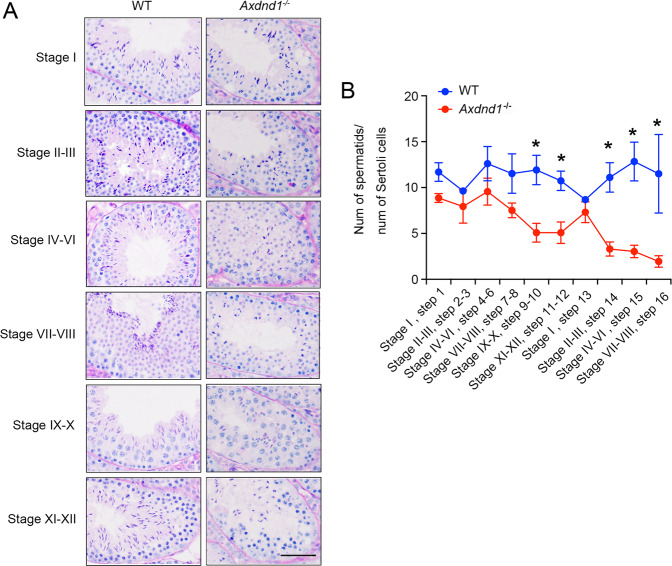
*Axdnd1*^*−/−*^ mice display progressive germ cell loss during spermiogenesis. **A** Representative images of seminiferous tubules at various stages from adult WT and *Axdnd1*^*−/−*^ male mice are shown. Disorganized germ cells and reduced late spermatids were observed in *Axdnd1*^*−/−*^ male mice. Scale bar: 100 µm. **B** Statistical analysis of the ratio of spermatids to the number of Sertoli cells during spermiogenesis (from steps 1–16). Significant loss of step 9–16 spermatids occurred in *Axdnd1*^*−/−*^ male mice during the later-half phase of spermiogenesis. Data are presented as Mean ± SEM, *n* = 3. *P < 0.05 by Student’s *t*-test.

To investigate the reason for infertility of *Axdnd1*^−/−^ male mice, we examined the morphology of seminiferous tubules on different stages by PAS staining assays. Compared with WT control mice, a remarkable decrease in the number of elongating and elongated spermatids were observed in all stages of seminiferous tubules in *Axdnd1*^*−/−*^ mice (Fig. 4A), suggesting possible abnormality occurred in the spermiogenesis. Further statistical analysis of the number of spermatids during spermiogenesis showed that in *Axdnd1*^*−/−*^ male mice, the ratio of spermatids was significantly decreased from step 9 and then reduced progressively till step 16 (Fig. 4B). Compared with WT control mice, the number of step 16 spermatids in *Axdnd1*^*−/−*^ mice was greatly declined. These results implied that AXDND1 is involved in mouse spermiogenesis.

**Fig. 5:**
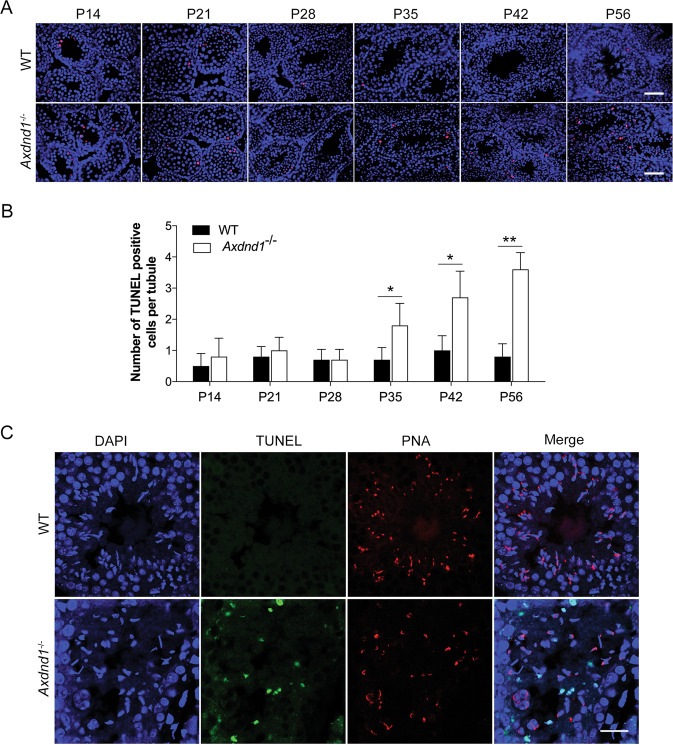
*Axdnd1*^*−/−*^ mice exhibit significantly increased apoptosis of late spermatids. **A** Representative images of the apoptosis of germ cells in developmental testes were determined by TUNEL staining (red). Scale bar: 50 µm. **B** Quantification of the number of TUNEL positive cells in (**A**). Data are presented as Mean ± SEM, *n* = 3. *P < 0.05 by Student’s *t*-test. **C** Co-staining of TUNEL and PNA in the adult testes from WT and *Axdnd1*^*−/−*^ mice. TUNEL (green), PNA (red), and DAPI (blue) were used to mark the apoptotic germ cells, acrosome, and nuclei, respectively. Scale bar: 20 µm.

To define whether the reduction of late spermatids was associated with apoptosis, TUNEL staining of mouse testes at different ages was performed. Compared with the WT mice, the results showed that significant apoptosis (red signals) into *Axdnd1*^*−/−*^ mouse testes occurred from P35 and then increased in an age-dependent manner (Fig. 5A, B). Further staining of adult mouse testis showed that TUNEL-positive (green) cells were mainly late spermatids located in the inner layer of seminiferous tubules, with acrosomes (PNA, red) could not be detected in these apoptotic spermatids (Fig. 5C). These results suggested that *Axdnd1* gene mutation in mice causes the late stage of spermatids undergoing apoptosis, thereby inducing late spermatids loss and male infertility.

### Impaired sperm head and flagellum development of late spermatids in *Axdnd1*^*−/−*^ mice

**Fig. 6:**
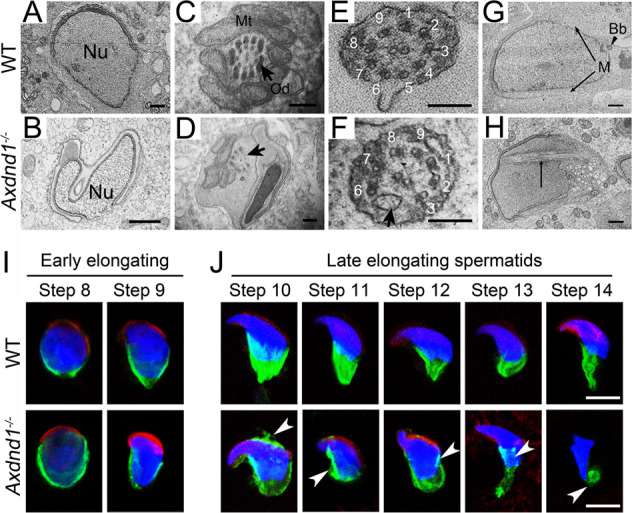
*Axdnd1* deletion in mice leads to abnormal spermatid development in late spermiogenesis. **A**–**H** Transmission electronic microscopy (TEM) analysis of testicular spermatids in WT and *Axdnd1*^*−/−*^ mice. A normal condensing sperm nucleus in WT elongating spermatids (step 8–9) is shown in (**A**) and an irregular-shaped sperm nucleus coupled malformed acrosome in *Axdnd1*^*−/−*^ elongating spermatids is shown in (**B**). The cross-section of flagella in WT (C and E) and *Axdnd1*^*−/−*^ (**D** and **F**) elongated spermatids are shown. Normal manchette located on the two sides of girdles of the nucleus in the WT elongating spermatid (**G**) and extra manchette microtubules appeared in the nucleus of *Axdnd1*^*−/−*^ elongating spermatid. Nu, nucleus; Mt, mitochondria; Od, outer dense fiber; Bb, basal body; M, manchette. Scale bar: 1 µm in (**A**–**B**), 200 nm in (**C**–**F**), 100 nm in (**G**–**H**). **I** Representative co-immunofluorescent staining images of EB3 (green) with PNA (red) in early elongating spermatids (step 8–9) between WT and *Axdnd1*^*−/−*^ are shown. **J** Representative co-immunofluorescent staining images of EB3 (green) with PNA (red) in late elongating spermatids (step 10–14) between WT and *Axdnd1*^*−/−*^ are shown. Residual manchette microtubules in the acrosome, as well as rigid and disorganized manchette microtubules (labeled by white arrowheads), were observed in step 10–14 spermatids of *Axdnd1*^*−/−*^ mice. Scale bar: 5 µm.

To determine the development of late spermatids in *Axdnd1*^*−/−*^ mice, we carried out transmission electron microscope (TEM) analysis of testicular tissues to investigate the ultrastructure of spermatids in *Axdnd1*^*−/−*^ mice. The results showed that a normal condensing sperm nucleus was observed in WT elongating spermatids (step 8–9) (Fig. 6A), but irregular-shaped sperm nucleus coupled malformed acrosome was found in *Axdnd1*^*−/−*^ elongating spermatids (Fig. 6B). In addition, the flagellum in WT elongated spermatids have a well-defined mitochondrial sheath, enveloping with typical “9 + 2” microtubules (Fig. 6C), whereas the flagellum in *Axdnd1*^*−/−*^ late spermatids displayed a partially lack of mitochondrial sheaths, along with incomplete ODFs and outer doublets (Fig. 6D). Detailed analysis showed that nine complete outer doublets were observed in WT flagellum (Fig. 6E), but the absence of the outer doublets 4–5 in the axoneme was frequently found in *Axdnd1*^*−/−*^ flagellum (Fig. 6F). Unlike laying on the two sides that girdle the nucleus during spermatid elongation in WT mice (Fig. 6G), extra manchette microtubules appeared in the nucleus of late spermatids in *Axdnd1*^*−/−*^ mice (Fig. 6H), suggesting an abnormality in manchette formation during spermiogenesis.

### Abnormal dynamics of manchette in *Axdnd1*^*−/−*^ spermatids

It was reported that proper dynamic change of manchette was required for the head shaping and sperm flagellum formation during spermiogenesis [13]. The sperm head and flagellum deformation in late spermatids of *Axdnd1*^*−/−*^ mice might probably be due to improper behavior of the manchette during spermatid elongation. To test this hypothesis, we isolated the round and elongated spermatids by BSA sedimentation and detected the dynamic change of manchette in these spermatids through immunofluorescent staining. EB3 (green) was a microtubule plus-end tracking protein and was usually used as a marker of manchette [3]. PNA (red) was used to label the acrosome. In the early elongating spermatids (step 8–9), we observed a discernable manchette structure formation in both WT and *Axdnd1*^*−/−*^ mice (Fig. 6I). Interestingly, in the late elongating spermatids (step 10–14), the well-organized manchette microtubules in WT mice began to migrate towards the flagellum, along with the condensing and elongation of the nuclei whereas the manchette microtubule structure in *Axdnd1*^*−/−*^ mice displayed abnormalities (Fig. 6J). In detail, although the migration of manchette microtubules toward the flagellum was observed, redundant manchette microtubules in the anterior region of the sperm head and disorganized, rigid, or discontinuous microtubules girdling the posterior end of the nucleus could be detected in *Axdnd1*^*−/−*^ mice (Fig. 6J). Importantly, we also observed AXDND1 colocalized with α-TUBULIN (a manchette marker) in human testes (Fig. 1C). Together, these results support a role for AXDND1 in manchette dynamic formation during late spermatid differentiation.

## Discussion

For the first time, the current study identified *Axdnd1* as a novel testis-specific gene in humans and mice that plays an essential role in male fertility. As most functional testis-enriched genes, the expression pattern of AXDND1 hinted at its function in spermiogenesis. In mouse testis, prominent expression of *Axdnd1* occurred from P21 onwards, when the round spermatids appear and sperm flagellum formation begins. Co-localization of AXDND1 and α-TUBULIN in human testis further raised this possibility. Further in vivo loss of function study showed that *Axdnd1* gene knockout in mice resulted in progressive germ cell loss, deformed sperm head and/or flagellum structures during the last stage of spermatogenesis, thereby causing male infertility.

Since no functional study has been reported about the *Axdnd1* gene, we generated the *Axdnd1* gene mutation mouse line by CRISPR/Cas9 and found *Axdnd1* mutated male mice were infertile in this study. During phenotypical analyses of spermatids via the gradient BSA, one notable thing is that only a few spermatids could be collected from the top elongated sperm-enriched fraction, which is consistent with the remarkably decreased amount of step 16 spermatids analyzed statistically after PAS staining. Further analysis of these separated germ cells showed that spermatids with various head deformities could be observed, while round spermatids seemed not affected. TEM results also showed that ill-shaped acrosomes surround the deformed sperm heads, some even with the incomplete nuclear envelope. According to the literature, the abnormal sperm head was usually related to a failure to properly form the acrosome or manchette, sometimes caused by improper nucleoprotein reorganization and chromatin condensation [19–22]. Abnormal acrosome was reported to be associated with the disordered distribution of spermatids, consistent with the irregular organization of germ cells in the *Axdnd1*^*−/−*^ male mice as revealed by PAS staining. In addition, we found AXDND1 could be colocalized with α-TUBULIN (a manchette marker) in human spermatids, suggesting AXDND1 is localized in the manchette and might be involved in manchette dynamic formation. Therefore, based on the results from the current study and literature reports, we inferred that AXDND1 deficiency affected the manchette function, which resulted in the ill-formed sperm head, abnormal acrosome, and subsequently malformed spermatids. However, the underlying molecular mechanism of how AXDND1 regulates manchette formation and function during spermiogenesis needs to be further investigated.

It is proposed that in the axoneme, doublets 4–7 facilitate microtubule sliding and are essential for sperm motility [23, 24]. Studies using mouse models revealed that deletion of *Ttll9*, *Vdac3*, *Dnah17*, or *Cfap97d1* resulted in the instability of sperm microtubule doublets 4–7, associated with sperm motility defects and male infertility [24–27]. Similarly, the ultrastructure analysis of testes from *Axdnd1*^*−/−*^ mice showed frequent loss of outer microtubule doublet 5–6 in the axoneme, which might be why the very few malformed sperm found in the epididymis were immotile. However, *Dnah17 and Cfap97d1* were MMAF (multiple morphological abnormalities of the sperm flagella) syndrome-associated genes, whose deficiency only caused defects when the sperm flagellum is assembled and undergoes elongation, but sperm heads were normal. Therefore, the mechanisms underlying might be different between AXDND1 and those MMAF related genes. Although the reasons why doublets 5–6 were absent in the *Axdnd1*^*−/−*^ sperm flagellum are still unknown, one hypothesis is that the deletion of AXDND1 affected the proper movement of manchette microtubules, which is responsible for the transport of proteins essential for sperm tail assembly.

In addition, previous studies have investigated the function of several IFT genes such as *Ift25*, *Ift27*, *Ift20*, *Ift172*, *Ift81* in knockout mouse models, and found these genes all play an essential role in sperm flagella formation during spermiogenesis [28–32]. Among these mouse models, the phenotype of *Ift25* knockout male mice shared the highest similarity with the *Axdnd1*^*−/−*^ male mice. They both showed phenotypes resembling severe oligoasthenoteratozoospermia (OAT), including remarkably low sperm concentration, immotile sperm with multiple morphological abnormalities in head and flagella [30]. But the abnormalities of the sperm flagellum in *Ift25*^*−/−*^ male mice were more versatile, including missing or disorganized axoneme microtubules and accessory structures (ODF or fibrous sheath) and distorted membranes, suggesting they may not function through the same mechanism. The complex interaction network of AXDND1 still needs further investigation.

In summary, our study has documented a previously uncharacterized gene and demonstrated that *Axdnd1* is essential for male fertility, specifically, the head shaping and sperm flagellum assembly during spermiogenesis, via affecting the manchette. These results will provide new insights into the mechanism underlying spermiogenesis as well as a potential target for clinical diagnosis and treatment for severe OAT.

## Materials and methods

### Mutation screening

Massively parallel sequencing had been performed to screen for variations in 654 genes potentially associated with NOA, including *AXDND1* (*C1ORF125*) [33]. 757 NOA patients were recruited from the Center of Reproductive Medicine, Tongji Medical College, Huazhong University of Science and Technology and 709 fertile men were recruited from the Center of Physical Examination, Peking University Shenzhen Hospital during January 2007 and October 2011. Collection of samples and exonic sequencing were approved by the ethics committee of Peking University Shenzhen Hospital and Tongji Medical College in accordance with the Declaration of Helsinki (Approval number: 20090018). All participant patients have signed the informed written consent to the research process. Both sequencing and data analysis was performed in Beijing Genomics Institute at Shenzhen, as described previously [33].

### Generation of *Axdnd1* knockout mice and FLAG-tagged *Axdnd1* mice

*Axdnd1*^*+/−*^ and *Axdnd1*^*FLAG/+*^ mice in C57/B6J background were established through CRISPR-Cas9 strategy from Cyagen company. Genotyping was performed using mouse tail genomic DNA, and PCR with primers as indicated (sequences were listed in Supplementary Table S2). All animals used in this study were maintained in SPF laboratory animal room and treated according to the Guide for the Care and Use of Laboratory Animals prepared by the Institute of Laboratory Animal Resources for the National Research Council. This study was approved by the ethics committee of Peking University Shenzhen Hospital and the animal center of Shenzhen PKU-HKUST Medical Center.

### Quantitative RT-PCR (RT-qPCR)

The details are provided in Supplementary Materials and Methods. Primers used to detect different transcripts were listed in Supplementary Table S2.

### Fertility test of mice

Sexually mature *Axdnd1*^*+/−*^ or *Axdnd1*^*−/−*^ male mice at about 8-weeks old were continuously caged with two 8-week-old female *Axdnd1*^*+/−*^, *Axdnd1*^*−/−*^ or WT mice for at least 3 months, respectively. The number of pups per litter was recorded. Average litter size was presented as the average number of pups from all the males tested.

### Histology of testis and epididymis

Human testis tissue was obtained from Alenabio company, China. Mouse testes and epididymis were dissected from adult WT and *Axdnd1*^*−/−*^ male mice and fixed in Bouin’s fixative or 4% paraformaldehyde (PFA) overnight. Details are provided in Supplementary Materials and Methods.

### TUNEL staining to assess apoptosis

TUNEL staining was performed with In Situ Cell Death Detection Kit, TMR Red (Roche, 12156792910) or In Situ Cell Death Detection kit, Fluorescein (Roche, 11684795910). Details are provided in Supplementary Materials and Methods.

### Isolation of testicular germ cells

To separate different types of germ cells in adult testis, gradient bovine serum albumin (BSA) sedimentation was performed as reported by Matteo with slight modification [34]. Details are provided in Supplementary Materials and Methods.

### Immunofluorescent staining

Testes were fixed in 4% PFA overnight at 4 °C and then were dealt with gradient sucrose and embedded in O.C.T. (Sakura Finetek, 4583) medium (50% OCT plus 10% sucrose). The detailed immunostaining procedures are provided in Supplementary Materials and Methods.

### Western blot

Tissue protein of adult male mice was extracted by RIPA lysis buffer and quantified with a BCA protein quantification kit (Pierce, 23225). The details are provided in Supplementary Materials and Methods.

### Transmission electron microscopy (TEM)

Testes were dissected from adult male mice and fixed immediately with 2.5% glutaraldehyde in PBS at 4 °C overnight. Then samples were embedded, cut into 70 µm-thick sections and then placed onto copper grids as described before [35, 36]. Images were taken with a Hitachi7100 TEM in Southern Medical University.

### Statistical analysis

Statistical analysis was performed using a student’s *t*-test. The *P*-value < 0.05 was considered statistically significant. Data were presented as Means ± SEM. Each experiment has been performed at least three times in three independent animals.

## Supplementary information

Supplementary Figure S1

Supplementary Figure S2

Supplementary Table S1

Supplementary Table S2

Supplementary materials

Author Contribution Statement

## Data Availability

All data needed to evaluate the conclusions in the paper are present in the article and/or the Supplementary Materials. All other supporting data of this study are available from the corresponding author upon reasonable request.
