# Supplementary figures and images for: AXDND1, a novel testis-enriched gene, is required for spermiogenesis and male fertility
Source: Cell Death Discov. 2021 Nov 11;7:348. doi: 10.1038/s41420-021-00738-z (PMC8580973; doi:10.1038/s41420-021-00738-z)

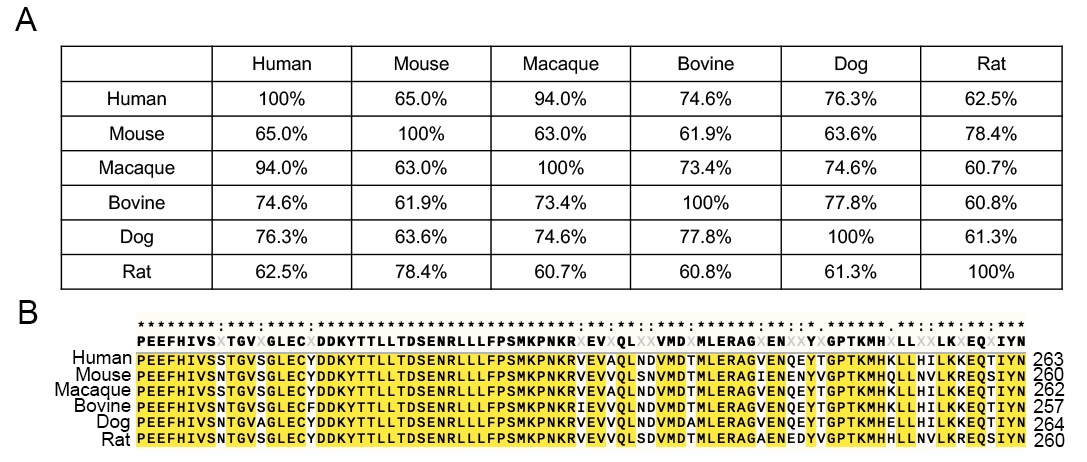

Supplement: Supplementary file 1 — Supplementary Figure S1 [file 41420_2021_738_MOESM1_ESM.jpg]

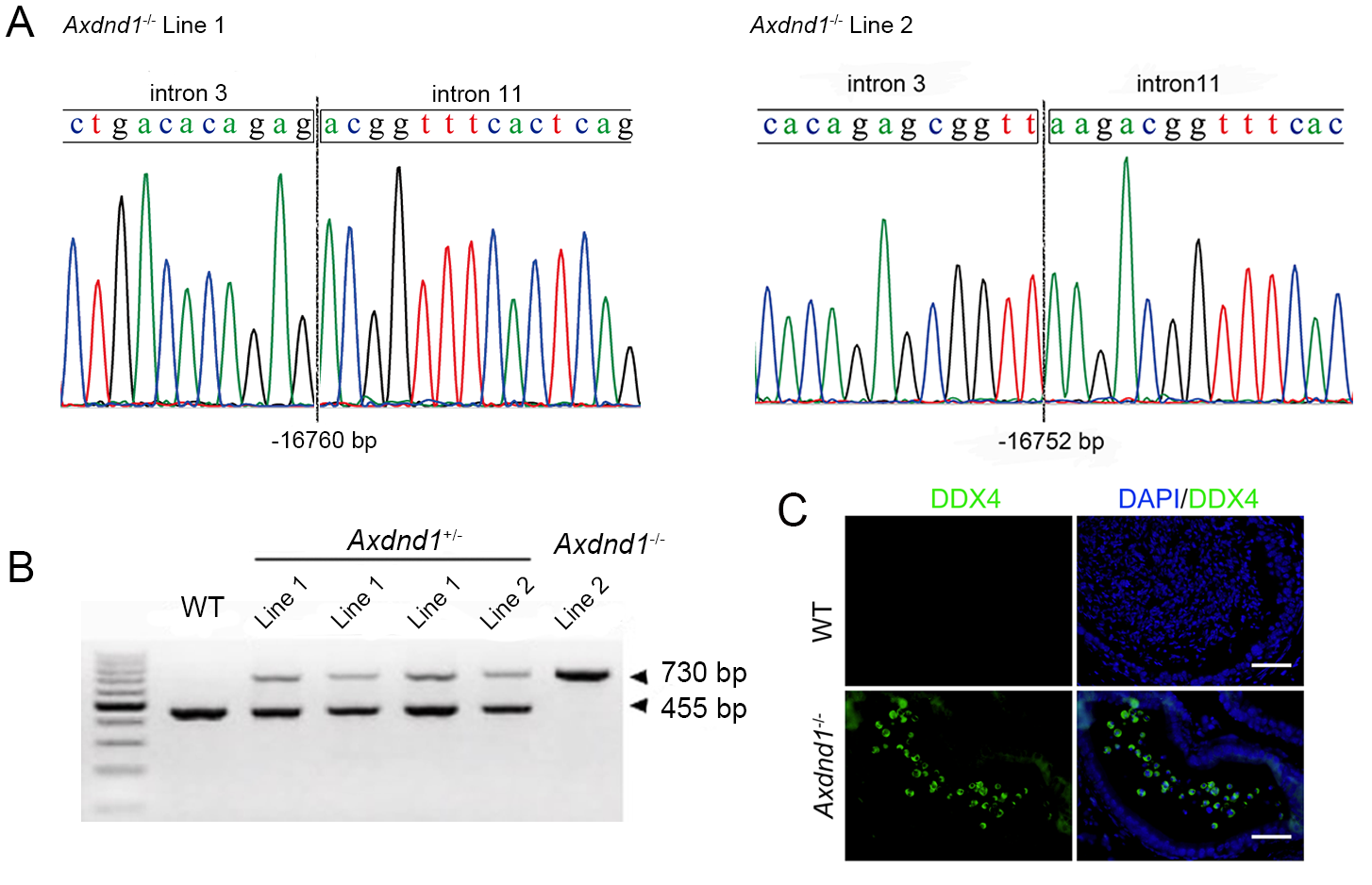

Supplement: Supplementary file 2 — Supplementary Figure S2 [file 41420_2021_738_MOESM2_ESM.tif]
