# Supplementary material for: AXDND1, a novel testis-enriched gene, is required for spermiogenesis and male fertility
Source: Cell Death Discov. 2021 Nov 11;7:348. doi: 10.1038/s41420-021-00738-z (PMC8580973; doi:10.1038/s41420-021-00738-z)
Supplement: Supplementary file 3 — Supplementary Table S1 [file 41420_2021_738_MOESM3_ESM.docx]

| cDNA  mutation | Protein  alteration | Function prediction | | |
| --- | --- | --- | --- | --- |
|  |  | SIFT | PolyPhen-2 | MutationTaster |
| c.244 C>T | p. P82S | Deleterious | Probably damaging | Diseasecausing |
| c.283 C>T | p. R95C | Deleterious | Probably damaging | Polymorphism |
| c.401 C>T | p. T134I | Neutral | Benign | Polymorphism |
| c.809 T>C | p. I270T | Deleterious | Probably damaging | Disease causing |
| c.984 T>A | p. H328Q | Neutral | Benign | Polymorphism |
| c.1304 A>G | p. K435R | Neutral | Benign | Polymorphism |
| c.1778 T>G | p. I593R | Deleterious | Benign | Disease causing |
| c.2483 G>A | p. R828Q | Neutral | Benign | Polymorphism |
| c.2774 T>C | p. I925T | Neutral | Possibly damaging | Polymorphism |

**Supplementary Table S1**. Identification and function prediction of 9 missense *AXDND1* variations unique to NOA patients are shown.
