# Supplementary material for: AXDND1, a novel testis-enriched gene, is required for spermiogenesis and male fertility
Source: Cell Death Discov. 2021 Nov 11;7:348. doi: 10.1038/s41420-021-00738-z (PMC8580973; doi:10.1038/s41420-021-00738-z)
Supplement: Supplementary file 4 — Supplementary Table S2 [file 41420_2021_738_MOESM4_ESM.docx]

**Supplementary Table S2**. Primer sequences used in this study.

| **Primers used for genotyping of *Axdnd1^+/-^* mice** | |
| --- | --- |
| F1 | GAGACTGTTCGCCAGGCTGTTG |
| F2 | ACATTTCCAGCATCACTAATAACAAGCT |
| R | TCTCTCAGCATCTTGTGGCTCATT |
|  | |
| **Primers used for genotyping of *Axdnd1^FLAG/+^* mice** | |
| F3 | CCTTGCTGCATAAGCCTTTATCTT |
| R3 | CAGCATCCTTTCTCATAAGCTGTT |
| F4 | TCTGTAGCGACCCTTTGCAG |
| R4 | ACAGGATGTCCCAGGCGAAG |
|  | |
| **Primers used for analysis of various isoforms of *Axdnd1* transcripts** | |
| *Axdnd1* 202/204 F | GTGGGGGAAAGGATGATGAT |
| *Axdnd1* 202/204 R | GACAGAACAATGGCCCGTG |
| *Axdnd1* 204 F | CAGGCGAATTATGGACAGCTTG |
| *Axdnd1* 204 R | GATGTTGATGGGGAGGTCAGC |
| *Axdnd1* 206 F | TGCTGCGTTGCTATGACGAC |
| *Axdnd1* 207 F | GTGGAATTATGGACAGCTTGAAAG |
| *Axdnd1* 206/207 R | CGAAGTGTCCGACACTTTGTG |
| *Axdnd1* 208 F | GCCAGAGAAGAGCCTGGGTT |
| *Axdnd1* 208 R | TAGAATTCAATCATCTGCTGGGC |
| *Axdnd1* 210 F | GTGTGTGACATGTTGAGACTCTGTC |
| *Axdnd1* 210 R | CATCGGTATCATCATCAATAGGAG |
| *Axdnd1* 211 F | GATTGGATCAGCACATGTTCAC |
| *Axdnd1* 211 R | CAATGGTGCTTCCTTTTCTTG |
| *Gapdh* F | AACTTTGGCATTGTGGAAGG |
| *Gapdh* R | ACACATTGGGGGTAGGAACA |
