# Supplementary material for: AXDND1, a novel testis-enriched gene, is required for spermiogenesis and male fertility
Source: Cell Death Discov. 2021 Nov 11;7:348. doi: 10.1038/s41420-021-00738-z (PMC8580973; doi:10.1038/s41420-021-00738-z)
Supplement: Supplementary file 5 — Supplementary materials [file 41420_2021_738_MOESM5_ESM.pdf]

Supplementary material for

AXDND1, a novel testis-enriched gene, is required for spermiogenesis and male fertility

Qian Ma, Congcong Cao, Changshui Zhuang, Xiaomin Luo, Xiaofeng Li, Huijuan Wan, Jing Ye, Fangfang Chen, Lina Cui, Yan Zhang, Yujiao Wen, Shuiqiao Yuan* and Yaoting Gui*

***Corresponding author Email:**

shuiqiaoyuan@hust.edu.cn

or

guiyaoting2007@aliyun.com

This PDF file includes:

Supplementary Figure S1. AXDND1 is highly conserved among all mammals.

Supplementary Figure S2. Generation of *Axdndn1* knockout mouse models by CRISPR/Cas9 technology.

Supplementary Table S1. Identification and function prediction of 9 missense *AXDND1* variations unique to NOA patients are shown.

Supplementary Table S2. Primer sequences used in this study.

Supplementary Materials and Methods.

A

	Human	Mouse	Macaque	Bovine	Dog	Rat
Human	100%	65.0%	94.0%	74.6%	76.3%	62.5%
Mouse	65.0%	100%	63.0%	61.9%	63.6%	78.4%
Macaque	94.0%	63.0%	100%	73.4%	74.6%	60.7%
Bovine	74.6%	61.9%	73.4%	100%	77.8%	60.8%
Dog	76.3%	63.6%	74.6%	77.8%	100%	61.3%
Rat	62.5%	78.4%	60.7%	60.8%	61.3%	100%

B

```
        ********:***:****:**************************:**:**::***:******:**::*.******.**::**:**:***
        PEEFHIVSxTGVxGLECxDDKYTTLLTDSENRLLLFPSMKPNKRxEVxQLxxVMDxMLERAGxENxxYxGPTKMHxLLxxLKxEQxIYN
Human   PEEFHIVSSTGVSGLECYDDKYTTLLTDSENRLLLFPSMKPNKRVEVAQLNDVMDTMLERAGVENQEYTGPTKMHKLLHILKKEQTIYN 263
Mouse   PEEFHIVSNTGVSGLECYDDKYTTLLTDSENRLLLFPSMKPNKRVEVVQLSNVMDTMLERAGIENENYVGPTKMHQLLNVLKREQSIYN 260
Macaque PEEFHIVSSTGVSGLECYDDKYTTLLTDSENRLLLFPSMKPNKRVEVAQLNDVMDTMLERAGVENQEYTGPTKMHKLLHILKKEQTIYN 262
Bovine  PEEFHIVSNTGVSGLECFDDKYTTLLTDSENRLLLFPSMKPNKRIEVVQLNDVMDTMLERAGVENQEYTGPTKMHKLLHILKKEQTIYN 257
Dog     PEEFHIVSNTGVAGLECYDDKYTTLLTDSENRLLLFPSMKPNKRVEVVQLNDVMDAMLERAGVENQEYTGPTKMHELLHILKKEQTIYN 264
Rat     PEEFHIVSNTGVSGLECYDDKYTTLLTDSENRLLLFPSMKPNKRVEVVQLSDVMDTMLERAGAENEDYVGPTKMHHLLNVLKREQSIYN 260
```

Supplementary Figure S1. AXDND1 is highly conserved among all mammals. (**A**) Amino acid sequence similarities of AXDND1 among 6 mammalian species. (**B**) Multi-alignment analyses of the amino acid sequence of AXDND1 in 6 mammalian species.

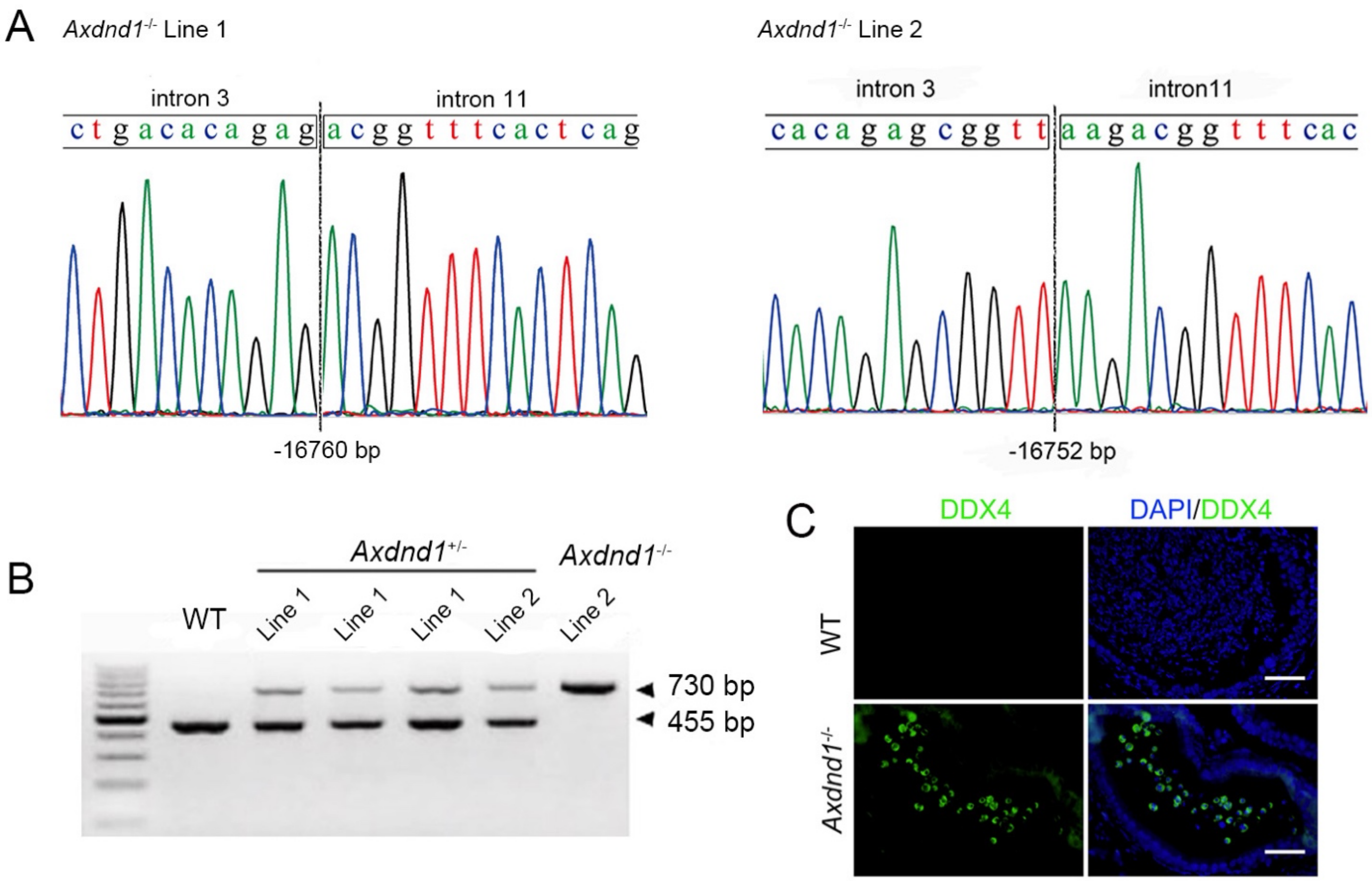

Supplementary Figure S2. Generation of *Axdndn1* knockout mouse models by CRISPR/Cas9 technology. (**A**)Two lines of *Axdnd1* gene knockout mice were generated. (**B**) Genotyping of two lines of *Axdnd1* gene knockout mouse models by PCR analyses. (**C**) Representative images of immunostaining with DDX4 (green) in caudal epididymis from WT and *Axdnd1*$^{-/-}$ mice. Scale bar: 50 μm.

Supplementary Table S1. Identification and function prediction of 9 missense *AXDND1* variations unique to NOA patients are shown.

cDNA mutation	Protein alteration	Function prediction		
		SIFT	PolyPhen-2	MutationTaster
c.244 C>T	p. P82S	Deleterious	Probably damaging	Disease causing
c.283 C>T	p. R95C	Deleterious	Probably damaging	Polymorphism
c.401 C>T	p. T134I	Neutral	Benign	Polymorphism
c.809 T>C	p. I270T	Deleterious	Probably damaging	Disease causing
c.984 T>A	p. H328Q	Neutral	Benign	Polymorphism
c.1304 A>G	p. K435R	Neutral	Benign	Polymorphism
c.1778 T>G	p. I593R	Deleterious	Benign	Disease causing
c.2483 G>A	p. R828Q	Neutral	Benign	Polymorphism
c.2774 T>C	p. I925T	Neutral	Possibly damaging	Polymorphism

Supplementary Table S2. Primer sequences used in this study.

**Primers used for genotyping of *Axdnd1*$^{+/-}$ mice**	
F1	GAGACTGTTCGCCAGGCTGTTG
F2	ACATTTCCAGCATCACTAATAACAAGCT
R	TCTCTCAGCATCTTGTGGCTCATT
**Primers used for genotyping of *Axdnd1*$^{FLAG/+}$ mice**	
F3	CCTTGCTGCATAAGCCTTTATCTT
R3	CAGCATCCTTTCTCATAAGCTGTT
F4	TCTGTAGCGACCCTTTGCAG
R4	ACAGGATGTCCCAGGCGAAG
**Primers used for analysis of various isoforms of *Axdnd1* transcripts**	
Axdnd1 202/204 F	GTGGGGGAAAGGATGATGAT
Axdnd1 202/204 R	GACAGAACAATGGCCCGTG
Axdnd1 204 F	CAGGCGAATTATGGACAGCTTG
Axdnd1 204 R	GATGTTGATGGGGAGGTCAGC
Axdnd1 206 F	TGCTGCGTTGCTATGACGAC
Axdnd1 207 F	GTGGAATTATGGACAGCTTGAAAG
Axdnd1 206/207 R	CGAAGTGTCCGACACTTTGTG
Axdnd1 208 F	GCCAGAGAAGAGCCTGGGTT
Axdnd1 208 R	TAGAATTCAATCATCTGCTGGGC
Axdnd1 210 F	GTGTGTGACATGTTGAGACTCTGTC
Axdnd1 210 R	CATCGGTATCATCATCAATAGGAG
Axdnd1 211 F	GATTGGATCAGCACATGTTCAC
Axdnd1 211 R	CAATGGTGCTTCCTTTTCTTG
Gapdh F	AACTTTGGCATTGTGGAAGG
Gapdh R	ACACATTGGGGGTAGGAACA

Supplementary Materials and Methods

Histology of testis and epididymis

Testis tissues were embedded in paraffin, sectioned into slides and stained with periodic acid-Schiff (PAS) or Hematoxylin and Eosin (HE), respectively. PAS staining was performed using a Kit (Solarbio, G1280) following the manufacturer's instructions. Briefly, slides were deparaffinized in xylene, rehydrated from ethanol to water, stained with PAS, counterstained with hematoxylin, dehydrated and cleared in xylene. HE was performed as described before. After sealing with neutral gum, images were captured with microscope (Olympus, BX53, Tokyo, Japan).

Quantitative RT-PCR (RT-qPCR)

Total RNA from tissues of wild-type adult male mice or from testes of mice aged 7, 14, 28, 35, 42, 49, 56 days was prepared with Trizol reagent according to the manufacturer's instructions. Then RNA was reverse transcribed into cDNA using the PrimeScript RT Master kit (Takara, RR037A). RT-qPCR was performed with the SYBR® Premix EX Taq™ II PCR Kit (Takara, DRR041A) following the manufacturer's instructions on the Roche Lightcycler 480 Real-Time PCR System. Data were calculated according to the Applied Biosystems comparative *Ct* method.

TUNEL staining to assess apoptosis

Briefly, testes sections were dewaxed and dehydrated, then treated with 20 μg/ml Proteinase K in 10 mM Tris-HCl (pH 7.5) for 15 min and incubated with TUNEL reaction mixture for 1 h at 37°C. The nuclei were stained with DAPI. Images were obtained with a fluorescent microscope (Olympus BX53, Tokyo, Japan) or a confocal microscope (Zeiss, LSM710, Germany).

Isolation of testicular germ cells

Testes from WT or *Axdnd1*$^{-/-}$ adult male mice were dissected and decapsulated in Krebs buffer. Collagenase Type Ⅳ (Sigma-Aldrich, V900893, final concentration of 1.0 mg/mL) was added to the tubules and incubated at 37°C for 5 min with gentle agitation. The separated tubules were washed with Krebs buffer twice and followed by digestion with trypsin (Gibco, 15090046, final concentration of 0.6 mg/mL) and DNase Ⅰ (Sigma-Aldrich, DN25, 10 ku/mL) at 37°C for 20 min with periodic vigorous agitation. The cells were filtered through a 40 μm cell strainer and pelleted by centrifugation at 600 g for 5 min at 4°C. After washing with Krebs buffer twice, dissociated cells were filtered, loaded onto a BSA gradient column composed of 5 %, 4 %, 3 %, 2 % and 1 % BSA, and gravity sedimented for 2

hours. Fractions enriched in elongating, round spermatids were collected and washed with Krebs buffer. Then cells were fixed in 4 % PFA and subjected to immunofluorescent staining as described.

Immunofluorescent staining

5μm cryo-sections were cut and subjected to antigen retrieval in a microwave oven with 0.01 M sodium citrate buffer (pH=6.0). Non-specific antibody binding sites were blocked with 5 % BSA for at least 30 min at room temperature. Primary antibodies were diluted in PBS and incubated at 4°C overnight. The primary antibodies used were rabbit pAb AXDND1 antibody (Invitrogen, PA5-64451, 1:200), rabbit mAb EB3 antibody (Abcam, ab157217, 1:250), or mouse mAb α-TUBULIN antibody (Invitrogen, 322588, 1:1000). Slides were then washed and incubated with secondary antibodies including donkey anti-mouse IgG-Alexa Fluor 488 (Invitrogen, A21202, 1:1000) or donkey anti-rabbit IgG-Alexa Fluor 594 (Invitrogen, A21207, 1:1000) for 2 hours at room temperature. PNA conjugated to Alexa Fluor 488 or 594 (Invitrogen, L21409 for 488 or L32459 for 594, 1:300) was used to mark acrosome during spermiogenesis. After three washes, testis sections were counterstained and mounted with DAPI-containing Vectashield (Vector Laboratories, H-1200) and observed under confocal microscopy (Zeiss, LSM710, Germany).

Western blot

Equivalent amount of protein from multiple tissues was subjected to SDS-PAGE, transferred onto PVDF membrane, blocked with 5 % non-fat milk and incubated with mouse mAb FLAG antibody (Sigma, F1804, 1:1000) at 4°C overnight. Then the membrane was incubated with HRP-conjugated goat anti-mouse secondary antibody (Abcam, ab6789, 1:10000) at room temperature for 2 h. Positive bands were detected by a Chemiluminescent kit (Millipore, WBKLS0500).
