# Supplementary material for: AXDND1, a novel testis-enriched gene, is required for spermiogenesis and male fertility
Source: Cell Death Discov. 2021 Nov 11;7:348. doi: 10.1038/s41420-021-00738-z (PMC8580973; doi:10.1038/s41420-021-00738-z)
Supplement: Supplementary file 6 — Author Contribution Statement [file 41420_2021_738_MOESM6_ESM.docx]

**Author Contribution Statement**

S.Y. and Y.G. conceived this study and supervised the project. Q.M., C.C., H.W. C.Z., X.L., X.L., Y.Z. and Y.W. performed histological analysis, immunofluorescent staining, TEM and all animal work. J.Y. performed data analysis. F.C. and L.C. performed RT-qPCR analysis. Q.M. wrote the manuscript. S.Y. revised the manuscript.
